# Social and environmental determinants of health among children with long-term movement impairment

**DOI:** 10.3389/fresc.2022.831070

**Published:** 2022-08-11

**Authors:** Ilene L. Hollin, Bethney Bonilla, Anita Bagley, Carole A. Tucker

**Affiliations:** ^1^Department of Health Services Administration and Policy, Temple University College of Public Health, Philadelphia, PA, United States; ^2^Center for Healthcare Policy and Research, University of California, Davis, Davis, CA, United States; ^3^Clinical Research, Shriners Hospitals for Children, Northern California, Sacramento, CA, United States; ^4^Department of Nutrition, Metabolic and Rehabilitation Sciences, University of Texas Medical Branch, School of Health Professions, Galveston, TX, United States

**Keywords:** social determinants of health, environmental determinants of health, pediatrics, movement impairments, functioning, participation, disability, neighborhood-level socioeconomic disadvantage

## Abstract

The healthcare research community increasingly recognizes the need to address social (SDOH) and environmental determinants of health (EDOH) to optimize health and healthcare. This is particularly relevant to disability and functioning and to those with child onset conditions that impair mobility and impact functioning and participation. Using the World Health Organization (WHO)'s International Classification of Functioning, Disability, and Health (ICF) as a comprehensive framework, this paper aims to discuss our understanding of the relationships between social and EDOH and outcomes among people with impaired mobility that impacts functioning. This paper offers suggestions for future developments and guidance to use SDOH and EDOH in research and clinical practice.

## Introduction

There has been broad recognition of the increasing need to address social determinants of health (SDOH) in healthcare (1–5). Efforts to better understand the role of SDOH from a theoretical perspective and early attempts to design interventions to address SDOH are emerging (6–13). Empirical research investigating the impact of SDOH on disability and functioning is in its nascent stages, and even less work has been done on this topic among pediatric populations. Children with long-standing movement impairments are susceptible to specific variations in environmental factors. Movement impairments may limit a child's ability to interact with their environment, may require the assistance of adaptations or technological devices, or may limit the ability to partake in leisure and recreation activities. Given the significant focus on SDOHs in recent years and their relevance for this population, we aim to provide a narrative review of the state of the science on SDOH and environmental determinants of health (EDOH) to better understand the impact of these determinants of health as they relate to individuals with child onset conditions that impact long-term mobility.

### Population

The population of interest in our work is individuals with long-term, child onset conditions that impair mobility and commonly impact an individual's functioning and participation in their physical and social environments. We focus on the United States because the measures of environmental factors are country specific. Child onset conditions that impact long-term mobility may be congenital or the result of an injury or disease. We define the population by ability (i.e., degree of impaired movement) rather than by condition because the source of the impairment is less relevant to our interest, which lies with the interaction between disability and functioning, and contextual factors. This approach to studying children by ability rather than diagnosis has been in use since the 1980s and assumes children and their families share similar experiences, challenges, and needs, regardless of the specific diagnosis (14–16). However, articulating categories of conditions and examples of conditions in those categories is useful for illustrative purposes. Common conditions that fall into this population of interest include, but are not limited to, the following: neuromuscular degenerative (e.g., spinal muscular atrophy, Duchene muscular dystrophy, or disorders of muscle mitochondria), neuromuscular non-progressive (e.g., cerebral palsy or spina bifida), skeletal/connective tissue (e.g., osteogenesis imperfecta, club foot, scoliosis, and limb deficiency), cognitive/intellectual developmental disability (e.g., Downs syndrome and atraumatic brain injury), major trauma (e.g., spinal cord injury, accidents, and amputations), neurosensory (e.g., blindness, epilepsy, hearing disorder, brachial plexus, and peripheral nerve injuries), and integumentary (e.g., third degree burns over more than 25% of the body, scleroderma, or epidermolysis bullosa). The proposed context of interest for our analyses spans all settings except acute care (e.g., in-patient hospitalization and intensive care).

Mobility impairment varies widely across the conditions listed above but also across children with the same condition. Factors that may contribute to this variation in mobility include the severity of the condition (e.g., gross motor function classification for cerebral palsy), the extent of limb involvement (e.g., level of spinal cord injury), and the number of systems involved (e.g., skeletal muscle and pulmonary in muscular dystrophy). Some of the conditions are progressive (e.g., muscular dystrophies). Others are not progressive, but symptoms may change with time and development (e.g., osteogenesis imperfecta and cerebral palsy). The presence of comorbid conditions that do not directly impact the movement system (e.g., asthma, obesity, and epilepsy) may worsen or complicate mobility impairments.

While it is difficult to define a general level of support, care, and treatment needed by this population, there are common needs of children with mobility impairments, which may involve medical treatment (e.g., surgery and pharmacological treatment), assistive devices for mobility (e.g., wheelchairs, crutches, and orthotics), and rehabilitative services, such as physical, occupational, or speech therapy. This population may require environmental modifications, caregiver support, or special accommodations at home and in the community to fully participate in their discretionary and required life roles. For instance, children with mobility impairments in a school setting commonly use assistive devices for mobility (e.g. leg braces, walkers, wheelchairs, etc.) and may need extra time moving around campus, special equipment, or adaptations in the classroom (e.g., positioning devices and adjustable desk heights), or help with specific class activities (e.g., a scribe to take notes or recording class lectures). Many of these adaptations are guided by local (e.g., school district, county), state, or federal policies and support systems.

The social factors and physical conditions of where people are born, live, learn, play, work, and age make up SDOH and EDOH. These factors impact a wide range of health, functioning, and quality-of-life outcomes. Examples of SDOH include the availability of resources to meet daily needs, such as educational and job opportunities, living wages, or healthy foods, social norms and attitudes, such as discrimination, exposure to crime, violence, social disorder, social support and social interactions, exposure to mass media and emerging technologies, such as the Internet or cell phones, socioeconomic conditions, such as concentrated poverty, quality schools, transportation options, public safety, and residential segregation (3). Similarly, systems of service, cultural and societal level beliefs, and expectations also influence health, and the critical impact of these SDOHs has become a recent focus of research. Environmental characteristics that influence health may include water quality, traffic, air pollution, toxins, weather, etc., and are often termed EDOH. The impact of long-term mobility impairments on an individual's function, activity, and participation in their physical and social environments warrants a deeper understanding of how SDOH and EDOH further influence their overall health, especially if we are to address those determinants through healthcare and public policy.

### Framework

To better understand the impact of social and EDOH on individuals with child onset conditions that impact long-term mobility, we will utilize the International Classification of Functioning, Disability, and Health (ICF) of the World Health Organization (WHO) (17). The ICF is one of the most comprehensive frameworks for understanding the relationships between SDOH and EDOH and provides a social model of disability, measuring health and disability at both the individual and population levels. As such, the framework allows for the discussion of the influence of the environment on participation (i.e., involvement in life situations). The ICF is based on a model of disability that incorporates both the medical model and the social model of disability and synthesizes the two into the biopsychosocial model (18). The ICF also provides a comprehensive life course perspective, inclusive of adult and child factors, making it appropriate for the discussion of child onset conditions that have implications on disability and functioning well into adulthood. Furthermore, the ICF aligns with our approach of defining the population by ability, functioning, and disability, rather than a diagnostic condition, emphasizing the interaction between the individual and their environment.

**Figure 1:**
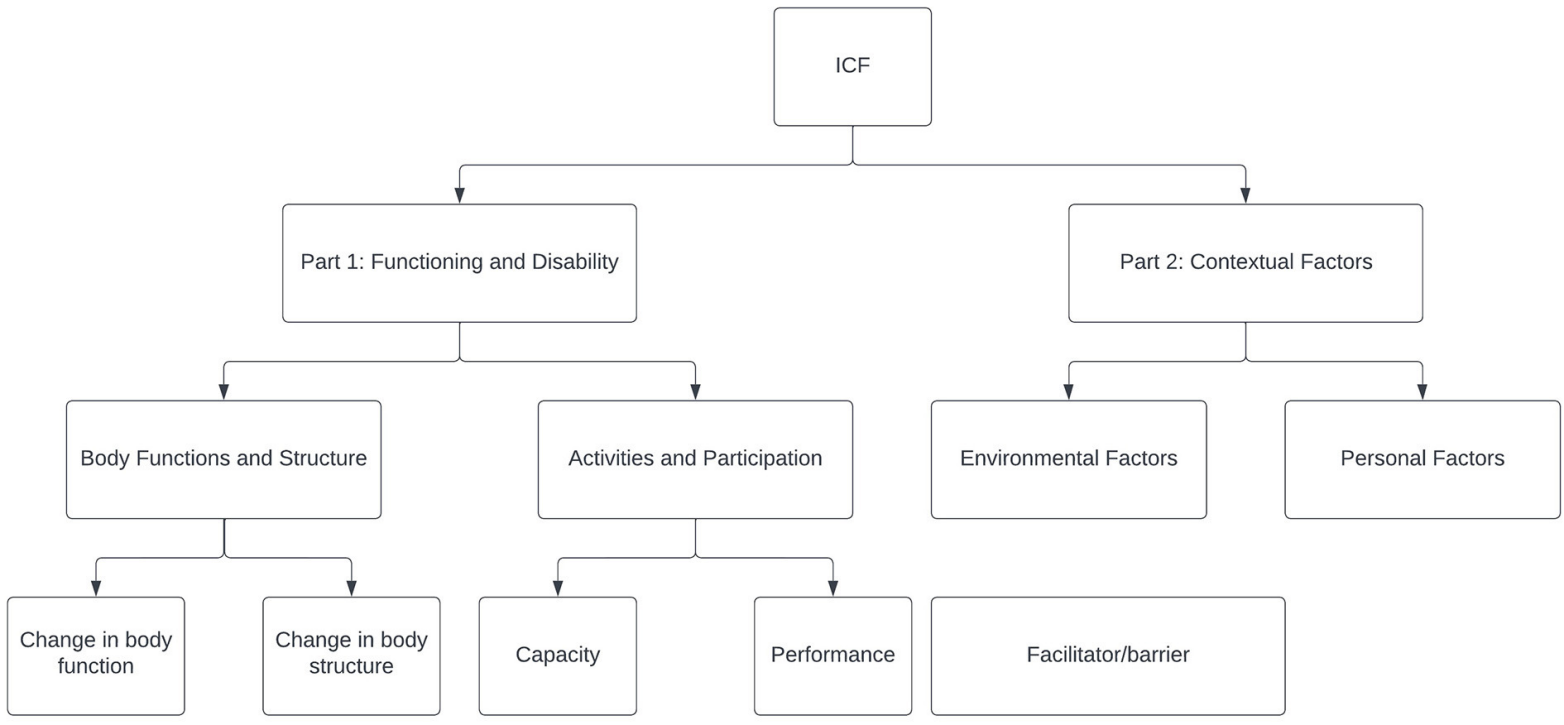
The International Classification of Functioning, Disability, and Health (ICF) framework (20).

The International Classification of Functioning, Disability, and Health model is a biospsychosocial model comprised of two parts: (1) functioning and disability and (2) contextual factors (see Figure 1) (18–20). The focus is on the interaction between those parts. Within functioning and disability, there are two components: (1.1) body functions and structures and (1.2) activities and participation. Within contextual factors, the components include both (2.1) environmental factors and (2.2) personal factors.

Environmental factors are contextual factors that are external or factors that are experienced at the community level, like socioeconomic composition and context, service provider attitudes about disability, and the availability of information regarding activities and programs (19). Environmental factors may also include social attitudes, architectural characteristics, legal and social structures, climate, terrain, etc. Within environmental factors, ICF sub-components include (1) products and technology, (2) natural environment and human-made changes to the environment, (3) support and relationships, (4) attitudes, and (5) services, systems, and policies.

Personal factors are contextual factors that are internal or factors that influence how disability is experienced at the individual level (19). Examples of personal factors include things about the home, parent's educational attainment, finances, supportive family relationship, and sensory qualities of the home (19). Examples of personal factors also include gender, age, coping styles, and social backgrounds (19). Other pieces of literature may refer to social needs instead of contextual factors and may differentiate between environmental factors and personal factors with the terms “community-level social needs” and “individual-level social needs” (1, 2, 19).

With a goal to improve functioning and participation among those with ability impairment, we must first understand how environmental factors impact functionality and participation. The difficulty lies in the measurement of environmental factors and combining those measures with other sources of information about personal factors, functioning, and ability. Functioning and ability are often characterized in electronic health records (EHRs), and to some extent, some personal factors are included there too. To improve general population health, others have called for the use of linked individual-level information and community-level SDOH and have identified the need to identify associations between the two and their combined impact on healthcare utilization and outcomes (1). It is unclear whether community-level SDOH does a better job at risk prediction than EHR data alone (1, 7, 11, 21–23). To further explore this for people with child onset mobility impairment, the field needs to understand available options for assessing environmental factors. We will use ICF as a framework for exploring some options for measuring social and physical environmental factors and for exploring ways to combine information about those factors with other data.

## Interactions between contextual factors and functioning and disability

Body functioning pertains to bodily functions related to all the body systems. Our focus is primarily on neuromuscular and movement-related functions, and our discussion of changes in the body structure is focused on structure related to movement. In the following section, we explore the ways in which neuromuscular and movement-related functions and structures interact with contextual factors.

### Personal factors, functioning, and disability

Race and ethnicity are personal factors that have been shown to be risk factors for conditions in which neuromuscular and movement-related functioning are affected. For instance, the prevalence of cerebral palsy (CP) is the highest among black infants and children (24–26). Black children also experience more traumatic brain injury (TBI)-related hospitalizations and have higher TBI-related mortality than white children (24, 27). Mexico-born women in the US are twice as likely to have a pregnancy with neural-tube defects, leading to spina bifida, perhaps due to unfortified diets (24, 28).

Socioeconomic status is (SES) another personal factor. A study in one region of the UK found a strong socio-economic gradient for CP in the normal birthweight category (although within the low and very low birthweight groups, there was no relationship between deprivation and CP prevalence) (29). Other studies have found that SES influences treatment outcomes for musculoskeletal conditions (30–38).

A retrospective chart review performed in one surgical practice assessed the role race, SES, and health insurance type play in healthcare outcomes for adolescent idiopathic scoliosis. They found that race impacted disease severity with black patients having higher mean curve magnitude (i.e., greater disease severity) and being more likely to present with curves in surgical range. Findings suggest that differences are due to race itself and not access to care or SES (39). A retrospective chart review assessing the role of insurance type, geographic SES, and ethnicity in adolescent idiopathic scoliosis in a state with mandated scoliosis screenings found that severity was not influenced by SES factors of ethnicity and household income (40).

### Environmental factors, functioning, and disability

Many environmental factors are dependent on the residence of a person. It has been shown that in cerebral palsy, where a child lives influences participation (41, 42). In another study looking at physical rehabilitation settings more generally, the author concludes that rehabilitation aftercare should be in close proximity to where an individual lives to promote empowerment of vulnerable social groups in burdensome living conditions (43).

The socioeconomic status (SES) of where a person lives is a contextual factor that is theorized to impact health (44). Community socioeconomic measures examine the social and economic conditions affecting the lives of all individuals who share a particular environment. Commonly used area measures include neighborhoods (variously described as ZIP codes, census tracts, census block groups, and census blocks) or other geographic areas (like counties, regions, and states) (45). Some studies have found that the socioeconomic conditions of a neighborhood may influence health independently of an individual's SES (46, 47).

There are a few studies that examine neighborhood SES and adult outcomes (30, 31, 48, 49). In the general pediatric healthcare space, low neighborhood SES is associated with worse perceived health across places and diagnoses. A cross-sectional study of pediatrics with upper extremity fractures in one orthopedic center found that children in the most disadvantaged neighborhoods had significantly worse mean patient reported outcome scores than those in the least disadvantaged neighborhoods (30).

Our population of interest, individuals with child-onset mobility impairments, demonstrate a wide range of person-environmental dynamics that are related to the interplay across multiple people and environmental (social and physical) factors. The balance between discretionary and non-discretionary life roles is complex, and the limits of this distribution have been recently amplified during the COVID pandemic. In children with movement abilities and their caregivers, the gaps in accessing necessary services, preferred activities, consistency and availability of external supports, and the time to live fully may seem nearly unattainable. For these families, access to coordinated care is key to reducing the gap, allowing families to participate fully in their life's roles. Coordinated care for children with mobility impairments focuses on short-term interventions directed at the individual's health assets (e.g., therapies to improve strength, walking, and functional activities), and environmental supports and adaptations to reduce challenges associated with the fit between the person and their environment (e.g., mobility aids, ramps, and augmentative communication devices). In addition to individual interventions, the critical needs are the means to change health system policies and ensure the integration of innovations derived from big data analytics to improve care. Finally, the transition from pediatric care settings to adult care settings presents additional challenges. For instance, the transition to adult care settings often lacks comprehensive coordinated care for child onset conditions during the transition, and the fragmented healthcare insurance system in the US often forces changes in insurance providers which can impact access to healthcare institutions and interrupt the continuity of care.

## Measurement of environmental factors

To further our understanding of the interaction between contextual factors and functioning and disability among children with long-term mobility impairments, empirical research studies are needed that measure environmental factors and combine those measures with measures of personal factors and measures of functioning. Then, empirical research studies will be needed that investigate how environmental factors modify the effect of treatment on health outcomes. Finally, once there is a better understanding of the impact of these environmental factors on health outcomes, we need to develop the means to incorporate these measures into routine clinical care.

Functional and disability outcomes are typically measured as part of clinical care and therefore are often readily available as part of a patient's medical record. Personal factors are contextual factors that are less often collected in routine clinical care as discrete data elements, but reflected in free text clinical notes in the patient's health record. Environmental factors, on the other hand, are less likely to be collected in clinical practice but may be accessible through publicly available data.

Although single measures (e.g., poverty rate) could be used to capture the socioeconomic characteristics of a community, researchers argue that a composite index made up of several key indicators drawn from domains like educational and occupational composition, income and employment distributions, or housing conditions, more accurately reflects the multidimensional characterization of a community's SES (50–53).

The area deprivation index (ADI) is a composite measure created by Singh in 2003 to measure socioeconomic disadvantage (50). Socioeconomic disadvantage is a concept that describes the individual level context or the neighborhood level context of low income, limited education, and substandard living conditions (54). The ADI ranks neighborhoods by socioeconomic disadvantage based on 17 US-based census poverty, education, housing, and employment indicators (50). It has been operationalized as a variable by defining levels of neighborhood disadvantage using percentile cutoffs for the national rank that range from 1 to 100 percentiles, or state rankings (1). The ADI is developed at the census block level and can be linked to a patient's home community using the 9-digit zip code (54).

The area deprivation index has been used to measure the impact of neighborhood socioeconomic disadvantage on the prevalence/incidence of various conditions, healthcare utilization and costs, care compliance, and quality improvement (1, 55–58). For example, Sheehy found 30-day re-observation among medicare beneficiaries, especially chronic re-observation, to be highly associated with neighborhood socioeconomic disadvantage, even after accounting for factors such as race, disability, and medicaid eligibility (58).

We are interested specifically in patient-reported outcomes from the Patient-Reported Outcomes Measurement Information System (PROMIS). To address the need for efficient (short), precise (reliable across a wide range of the latent trait), and valid measures of self-reported physical, mental, and social health that can be used in clinical research and practice, in 2004, the National Institutes of Health launched a program of research called the patient reported outcome measurement information system (PROMIS) (59). PROMIS measures provide psychometrically robust, pediatric patient-reported outcome measures across physical, social, and mental health domains that have undergone conceptual mapping and item-level mapping to the ICF (60–62). In addition, PROMIS measures have been deployed broadly across health care systems (e.g., Shriners Hospitals for Children) and embedded in EHR architectures (e.g., Epic). The PROMIS health organization (PHO) and healthmeasures.net continue to expand the rigorous PROMIS mixed-methods approach for developing instruments that assess the lived experiences of physical, mental, and social health (59, 60, 63). PROMIS has produced numerous measures that are applicable across the life course and are in widespread use internationally.

There are a few examples of the ADI being used to assess the relationship between patient-reported outcomes and treatment/clinical outcomes in various populations. The focus of such studies is usually on differences in patient-reported outcomes by socioeconomic status upon initial presentation. For instance, one such study of patients presenting to an orthopedic provided used the ADI to determine that patients living in zip codes with a higher disadvantage compared to those from less disadvantaged areas reported lower levels of physical functioning and higher levels of pain interferences, depression and anxiety as measured by PROMIS (64). In another study, neighborhood socioeconomic disadvantage was found to be correlated with function in pediatric patients with congenital hand differences; child self-reported PROMIS scores for pain interference, peer relations, anxiety, and depression were worse in more socially disadvantaged areas, suggesting more psychosocial challenges in these children (65). A third study evaluated the impact of a neighborhood socioeconomic disadvantage on PROMIS scores in children presenting for treatment of upper extremity fractures and found children living in areas of greatest socioeconomic disadvantage report worse upper extremity function, mobility, pain interference, and peer relations scores on self-administered PROMIS CATs than children from areas of least socioeconomic disadvantage (30). A follow-up study was conducted to determine whether this difference in outcome scores would resolve after children received orthopedic treatment for their fractures. While neighborhood socioeconomic disadvantage was not associated with any difference in the improvement in PROMIS scores from injury to healed fracture, the data showed that children living in areas of greater socioeconomic disadvantage reported worse pain and perceived function even after treatment (66).

Gaps in research using ADI to assess health outcomes exist. There is limited work in this area in general and no reports of this type for the population with childhood onset long-term mobility impairments.

The second measure of neighborhood disadvantage is the distressed communities index (DCI). Like the ADI, the DCI is also a composite score. The DCI is based on seven metrics of socioeconomic disadvantage and scores each zip code from 0 (no distress) to 100 (severe distress). The DCI has been used to highlight the impact of socioeconomic status on surgical outcomes (67).

There are options for measuring environmental factors other than a socioeconomic disadvantage. The Gini index, or coefficient, is a measure of income inequality that summarizes the dispersion of income across the entire income distribution (68). The Robert Wood Johnson County Health Rankings provide health rankings of individual counties within states using different sets of publicly available data (69). These data have been used to measure health differences between rural and urban areas across the US (70). However, there is a paucity of examples of these tools being used to measure environmental factors as it relates to health outcomes.

The design of composite measures must account for the weighting of their component indicators comprising the index, either explicitly or implicitly. For instance, the original design of the ADI applied a weighting schema such that poverty, income, and education were more heavily weighted than other components, whereas the DCI weights its components equally. When considering the use of general composite measures to the specific context of disability and functioning, more specifically to child onset conditions that impair mobility and impact functioning and participation, it is worth considering applying a custom weighting schema to the component indicators of the measures. To tailor measures in this way may make them more relevant in consideration of a particular context or disability but introduce additional data needs, specifically the component indicators in addition to the composite measure. Additional research would be required to better understand the relative importance of various component indicators in a particular context.

Developing a fit-for-purpose index of environmental factors is another possible option to advance our understanding of the relationship between environmental factors and functioning and mobility. A fit-for-purpose measure could incorporate multiple sources of publicly available data at the zip-code level, similar to the use of Census-derived data to calculate the ADI and DCI. The advantage to this personalization would be the ability to focus on factors theorized to be most relevant to functioning and mobility. The tradeoff of a fit-for-purpose measure is that it moves the research community away from a universal measure (11). A universal measure with relevance across a variety of contexts would generate more knowledge applicable across multiple settings and would more likely be merged with other data or incorporated into data systems.

## Potential developments

If disability and functioning are outcomes of interactions between health conditions (e.g., diseases, disorders, and injuries) and contextual factors, we need to account for both environmental contextual factors and personal contextual factors (19). The dynamics between an individual and his/her environments, between capacity, performance and participation, between available resources and lived challenges are often represented as a “gap.” The increased focus on addressing SDOH and EDOH to enhance health outcomes and reduce disparities provides a wealth of possible approaches to reduce this gap and improve health-related quality of life.

One environmental factor, neighborhood socioeconomic disadvantage, can be measured and has been associated with health outcomes at initial presentation for multiple conditions, but additional research is needed to determine if neighborhood disadvantage modifies the impact of treatment benefit, especially among children with long-term movement impairments. The short-term goal is to understand if children with increased neighborhood disadvantage start with a lower baseline of health and, if so, how does this impact the magnitude of improvement from treatment?

To achieve this goal, an initial step is to merge an existing measure of neighborhood socioeconomic status, such as ADI, with existing electronic health record data (i.e., zip code) to describe neighborhood disadvantage and its relationship to patient outcomes. A second step would be to improve the data we collect with the objective of tracking more contextual factors (1, 7). We could do this by embedding questionnaires and screening tools into electronic health records for contextual factors. To start, any effort to expand this type of data collected should focus on the identification of individual-level factors that can be included in the electronic health record because we can leverage existing data for community-level social variables that match zip code (71).

The long-term goal of these efforts is to be able to identify which children may be at higher risk for poor outcomes due to neighborhood socioeconomic disadvantage (and other social and environmental factors). This would lead to many real-world applications. It would enable the development of risk prediction models to help health systems identify children with risk factors that could impact health care utilization or health outcomes and intervene proactively (11, 72). An understanding of environmental factors at the point of care would help provide individual, context-specific health care. The identification of environmental barriers or challenges would help clinicians plan to help address factors that can impact medical management.

At the policy level, defining children at risk as a result of EDOH would help policymakers identify where to address specific community-level issues and develop interventions. Such data could impact housing policies, transportation availability and quality, employment policies, and the availability of programs and services for young children with disabilities and families (73). It could also report the design of risk-adjustment models for environmental risk factors in value-based care payment models (11). Proprietary tools are already being developed in the private industry and marketed to insurance companies for risk adjustments to premiums based on social determinants of health (74).

The proposed use of existing measures of neighborhood socioeconomic status to understand and address environmental factors and their impact on potential treatment is not without its limitations. First, neighborhood-level measurement of socioeconomic status may not be suitable on a global scale because of large inequalities within a neighborhood. We focus on the United States because the measures of environmental factors discussed utilize census data and therefore are specific to the US context. The Global Multidimensional Poverty Index (MPI), and similar global measures, may be considered in global or cross-national contexts (75). Second, these measures are intended to be objective and therefore do not account for an individual's experience of their community, or the legislative, attitudinal, and regulatory context within which the community sits (i.e., state or national level). To define the individual's experience within the community, surveys or qualitative data collection may be required (41, 76). To understand the state or national context, state or national comparisons are required. Subjective measures (*via* questionnaire) of the presence/absence of physical and attitudinal barriers in the community, family, and school environment (77–79) may be useful in a research context to inform and develop a more complete landscape of environmental factors. Attitudinal barriers that would be particularly useful in the context of mobility and functioning include beliefs around disability or stigma toward disability.

## Next steps and proof of concept

Our group's next step is establishing resources and linkages that will allow existing learning health systems, which are predominantly EHR data-driven, to integrate with databases of SDOH/EDOH. The patient centered outcomes research institute (PCORI) has identified the integration of SDOH/EDOH within its vast network of Research Data Networks. Our work continues to establish linking in a pediatric learning health system with the existing databases of SDOH/EDOH to provide families and clinicians such critical environmental information to improve patient care and outcomes. This integration of environmental knowledge with health record data allows the “gap” between the individual and their environment to be considered holistically and health interventions to address their interplay more thoughtfully.

## Conclusion

This narrative review describes the state of the science on SDOH and EDOH to better understand the impact of these determinants of health as they relate to individuals with child onset conditions that impact long-term mobility. We describe the limited empirical research that evaluates the impact of SDOH and EDOH on disability and functioning. We discuss why children with long-standing movement impairments are susceptible to specific variations in environmental factors. Finally, we suggest options for capturing specific measures of EDOH, such as neighborhood disadvantage, using existing data.

## Author contributions

CT conceptualized the work. IH drafted the article. BB, AB, and CT critically revised the article. All authors contributed to the design of the work and provided final approval of the version submitted for publication.

## Funding

The Shriners Health Outcomes and Wellness Network was funded by Shriners Hospitals for Children clinical research grant #79158 (PI: CT).

## Conflict of interest

The authors declare that the research was conducted in the absence of any commercial or financial relationships that could be construed as a potential conflict of interest.

## Publisher's note

All claims expressed in this article are solely those of the authors and do not necessarily represent those of their affiliated organizations, or those of the publisher, the editors and the reviewers. Any product that may be evaluated in this article, or claim that may be made by its manufacturer, is not guaranteed or endorsed by the publisher.

## Author disclaimer

The content presented here is solely the responsibility of the authors and does not necessarily represent the official views of the Shriners Hospital for Children.
